# Outpatient intravenous diuresis in a rural setting: safety, efficacy, and outcomes

**DOI:** 10.3389/fcvm.2023.1155957

**Published:** 2023-05-25

**Authors:** Girish Pathangey, Susan P D’Anna, Rohitha A. Moudgal, David B. Min, Katharine A. Manning, Cynthia C. Taub, Lauren G. Gilstrap

**Affiliations:** ^1^ Department of Medicine, Dartmouth-Hitchcock Medical Center, Lebanon, New Hampshire; ^2^ Heart and Vascular Center, Dartmouth-Hitchcock Medical Center, Lebanon, New Hampshire

**Keywords:** rural-urban, heart failure, rural health, rurality, health disparities, diuresis clinic, outpatient intravenous diuresis

## Abstract

**Purpose:**

To evaluate the safety, efficacy, and outcomes of outpatient intravenous diuresis in a rural setting and compare it to urban outcomes.

**Methods:**

A single-center study was conducted on 60 patients (131 visits) at the Dartmouth-Hitchcock Medical Center (DHMC) from 1/2021–12/2022. Demographics, visit data, and outcomes were collected and compared to urban outpatient IV centers, and inpatient HF hospitalizations from DHMC FY21 and national means. Descriptive statistics, T-tests and chi-squares were used.

**Results:**

The mean age was 70 ± 13 years, 58% were male, and 83% were NYHA III-IV. Post-diuresis, 5% had mild-moderate hypokalemia, 16% had mild worsening of renal function, and 3% had severe worsening of renal function. No hospitalizations occurred due to adverse events. The mean infusion-visit urine output was 761 ± 521 ml, and post-visit weight loss was −3.9 ± 5.0 kg. No significant differences were observed between HFpEF and HFrEF groups. 30-day readmissions were similar to urban outpatient IV centers, DHMC FY21, and the national mean (23.3% vs. 23.5% vs. 22.2% vs. 22.6%, respectively; *p* = 0.949). 30-day mortality was similar to urban outpatient IV centers but lower than DHMC FY21 and the national means (1.7% vs. 2.5% vs. 12.3% vs. 10.7%, respectively; *p* < 0.001). At 60 days, 42% of patients had ≥1 clinic revisit, 41% had ≥1 infusion revisit, 33% were readmitted to the hospital, and two deaths occurred. The clinic avoided 21 hospitalizations, resulting in estimated cost savings of $426,111.

**Conclusion:**

OP IV diuresis appears safe and effective for rural HF patients, potentially decreasing mortality rates and healthcare expenses while mitigating rural-urban disparities.

## Introduction

Heart failure (HF) in the United States poses a substantial economic strain on the healthcare system, with annual expenses surpassing $30 billion and an anticipated 50% surge in prevalence by 2030 (1). Despite treatment advancements, 30-day readmission and mortality rates have plateaued, approaching 25% and 10% respectively (2). One strategy to augment HF care and reduce readmission costs is the implementation of outpatient intravenous (OP IV) diuresis to address decompensated HF in outpatient clinics, infusion centers, and more recently, home-based IV diuresis care. While preliminary studies have demonstrated promising results in urban settings, additional research is necessary to assess the efficacy in rural communities (3–10).

Rural residents with HF encounter unique challenges such as higher rates of comorbidities, underinsurance, limited healthcare access, and increased travel distances (11, 12). Rural-urban disparities have widened, with rural hospitals experiencing up to 20% higher 30-day mortality rates for HF patients compared to non-rural hospitals (11). Considering that 20% of the American population resides in rural areas, it is important to investigate the potential benefits of OP IV diuresis for rural HF patients to alleviate hospital burden and bridge the gap in rural-urban disparities. This pilot study aims to evaluate the safety and effectiveness of a rural outpatient intravenous (OP IV) diuresis clinic, as well as the 30- and 60-day outcomes, hospitalizations avoided, and estimated cost savings.

## Methods

### Study design

**Figure 1:**
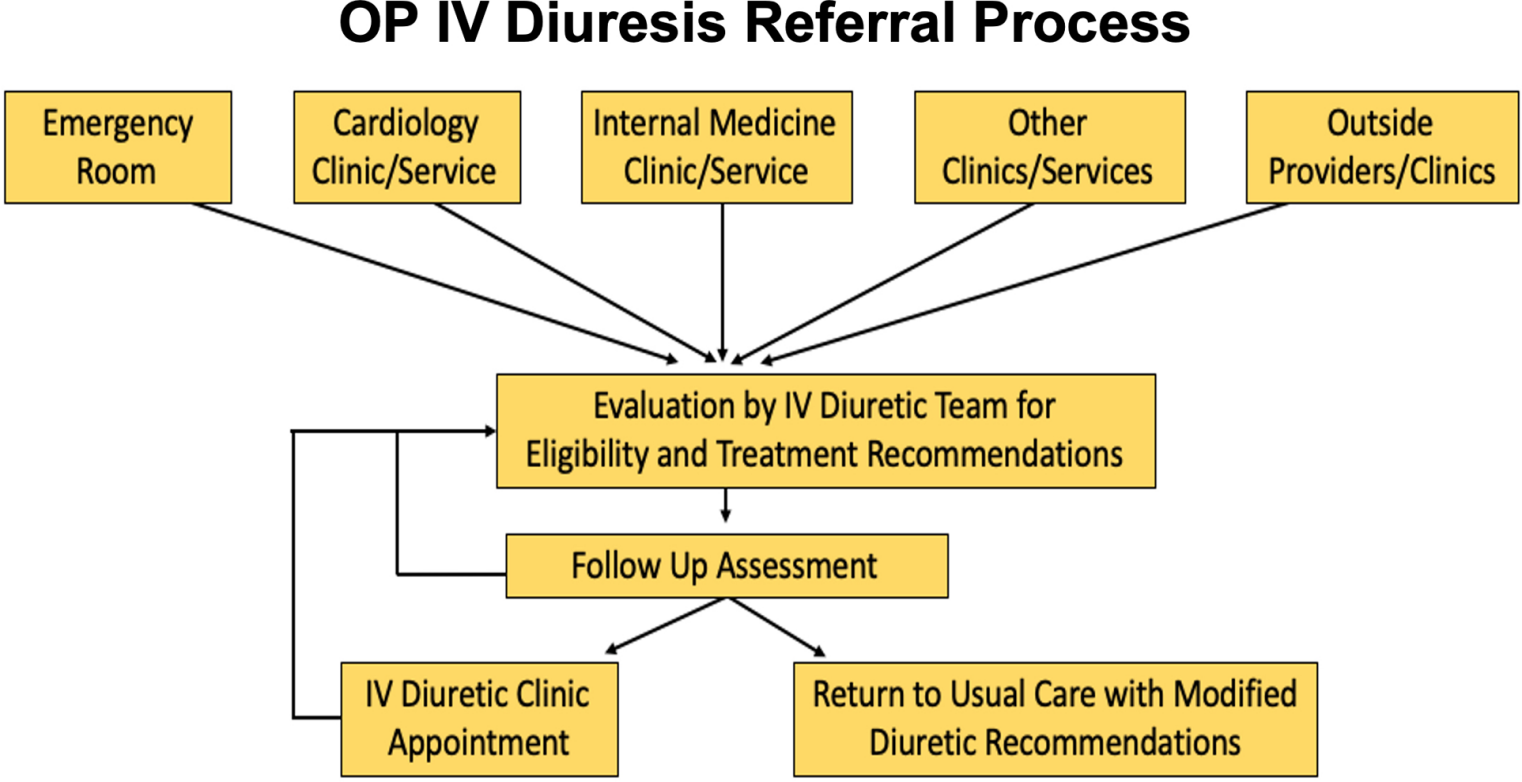
Patients are referred to the rural outpatient IV diuresis clinic from various sources, including the emergency department, inpatient services, and outpatient providers. Referrals are then assessed by the OP IV Diuresis Team for eligibility to continue with the treatment in the DHMC OP IV center (19).

A single-center, prospective study was conducted on 60 patients (131 visits) undergoing OP IV diuresis at Dartmouth-Hitchcock Medical Center (DHMC) from 1/2021–12/2022. Approved by the institutional review board and funded by the Levy Health Care Delivery Incubator grant, the study included patients referred from the emergency department, inpatient and outpatient services, and external providers for initial and/or additional IV diuresis, along with close follow-up monitoring (Figure 1). Eligibility criteria were modeled after CARRESS-HF trial and Buckley et al., which included participants with chronic heart failure experiencing worsening congestion and a resting systolic blood pressure above 90 mmHg (4, 13). Exclusion criteria comprised advanced chronic kidney disease or end-stage renal disease, secondary concerns related to acute decompensated heart failure, and severe symptoms accompanied by clinical instability. Baseline demographics and outpatient diuretic regimen were collected. The 2010 Rural-Urban Commuting Area classification system was used to categorize residential areas into four levels of rurality (14).

### Interventions

The clinic is staffed by advanced practice providers and registered nurses, supervised by a board-certified cardiologist. Prior to each infusion visit, basic metabolic panel is drawn, and IV access is obtained. The appropriate infusion regimen is determined based on patient's home diuretics, electrolytes, renal function, and blood pressure; infusion regimens include boluses, drip infusions over several hours, or a combination of both. The clinic exclusively offered short-term IV diuresis without modifying or adding to the patient's home guideline-directed medical therapy, such as ARNI and SGLT2i. During the visit, patients' weights are recorded, vitals are measured at 30 min intervals, telemetry is conducted, and access to ancillary services is provided. Follow-up phone calls are made 24–72 h post-diuresis for response.

### Outcomes

Safety outcomes were evaluated 24–72 h post-infusion for treatment-related hypokalemia and worsening renal function. Efficacy outcomes include urine output during infusion and weight loss post-infusion. Clinic and infusion revisits, hospitalizations, and all-cause mortality were evaluated at 30 and 60 days. 30-day outcomes were compared to urban OP IV diuretic centers and Medicare HF hospitalizations from DHMC FY21 and national means (3–10). Hospitalizations avoided were adjudicated by a minimum of two cardiologists. Total cost savings were calculated using hospitalizations avoided multiplied by the mean cost of DHMC HF hospitalization in FY21. Descriptive statistics, T-tests and chi-squares were performed using IBM SPSS Statistics (version 24). Post-hoc sample size calculations were conducted based on the observed significant primary outcome of rural OP IV diuresis compared to the population's national mean of HF hospitalization or to account for potential study attrition when compared to DHMC FY21 HF hospitalizations.

## Results

The mean age was 70 ± 13 years, and 58% were male. 79% had a Charlson Comorbidity Index score of ≥3 (Table 1). Before decompensation, the mean ejection fraction was 49 ± 16%, and 83% reported NYHA functional class III-IV. No significant differences were observed between HFpEF and HFrEF groups. 3% of patients came from urban areas, 37% from large rural areas, 33% from small rural areas, and 25% from isolated rural areas, and patients on average traveled 34 ± 25 miles to the clinic. 67% of the patients were on maintenance torsemide, 25% were on furosemide, and 13% were on metolazone in conjunction with loop diuretics.

**Table 1:** The mean age was 70 ± 13 years and 58% were male. Our patient population had advanced heart failure (83% NYHA III-IV) and high comorbidity burden (79% Charlson Comorbidity Index score ≥3), requiring high maintenance doses of diuretics. Despite higher rates of uncontrolled traditional cardiovascular risk factors in rural areas (11), the NYHA class and comorbidities of our patients were similar to those of urban OP IV centers (3–10). There were no significant differences observed between HFpEF and HFrEF groups. ACEi = Angiotensin-converting enzyme inhibitor; ARB = Angiotensin receptor blocker; MRA = Mineralocorticoid receptor antagonist; ANRI = Angiotensin receptor neprilysin inhibitor; SGLT2i = Sodium-glucose cotransporter 2 inhibitor.

	Total (*n* = 60)	HFpEF (*n* = 21)	HFrEF (*n* = 39)	*p*-value
**Demographics**				
Age	70 ± 13	68 ± 14	71 ± 13	0.456
Female	42% (*n* = 25)	48% (*n* = 10)	38% (*n* = 15)	0.507
Dry Weight	219 ± 64 (*n* = 55)	239 ± 70 (*n* = 19)	208 ± 59 (*n* = 36)	0.117
Distance	34 ± 25	36 ± 28	33 ± 24	0.612
Urban	3% (*n* = 2)	5% (*n* = 1)	3% (*n* = 1)	0.687
Large Rural	37% (*n* = 22)	33% (*n* = 7)	38% (*n* = 15)	0.699
Small Rural	33% (*n* = 20)	38% (*n* = 8)	33% (*n* = 13)	0.471
Isolated Rural	25% (*n* = 15)	24% (*n* = 5)	26% (*n* = 10)	0.097
**Medical History**				
Atrial Fibrillation	52% (*n* = 31)	38% (*n* = 8)	59% (*n* = 23)	0.129
Coronary Artery Disease	50% (*n* = 30)	43% (*n* = 9)	54% (*n* = 21)	0.427
Chronic Kidney Disease	48% (*n* = 29)	57% (*n* = 12)	44% (*n* = 17)	0.328
Diabetes	53% (*n* = 32)	57% (*n* = 12)	51% (*n* = 20)	0.671
Hypertension	68% (*n* = 41)	71% (*n* = 15)	67% (*n* = 26)	0.709
Hyperlipidemia	70% (*n* = 42)	80% (*n* = 17)	64% (*n* = 25)	0.157
COPD	18% (*n* = 11)	19% (*n* = 4)	18% (*n* = 7)	0.919
Pulmonary Hypertension	47% (*n* = 28)	62% (*n* = 13)	38% (*n* = 15)	0.088
Mod/Severe AS	24% (*n* = 12)	27% (*n* = 5)	22% (*n* = 7)	0.610
Mod/Severe MR	5% (*n* = 3)	–	8% (*n* = 3)	–
ICD or CRT	20% (*n* = 11)	10% (*n* = 2)	26% (*n* = 10)	0.077
**Risk Assessment**				
LVEF	49%±16	65%±4	39%±14	**< 0.001**
NYHA Class III-IV	83% (*n* = 50)	76% (*n* = 16)	87% (*n* = 34)	0.835
**Home Diuretic Regimen**				
Furosemide	25% (*n* = 15)	24% (*n* = 5)	26% (*n* = 10)	0.878
Torsemide	67% (*n* = 33)	76% (*n* = 16)	62% (*n* = 24)	0.242
Bumex	7% (*n* = 4)	–	10% (*n* = 4)	-
Metolazone	13% (*n* = 8)	24% (*n* = 5)	8% (*n* = 3)	0.134
Furosemide Dose	101 ± 55	128 ± 66	87 ± 45	0.256
Torsemide Dose	107 ± 61	106 ± 63	108 ± 60	0.951
Bumex Dose	3.3 ± 1	–	3.3 ± 1	–
Metolazone Dose	3 ± 1	3 ± 1	2.5 ± 0	0.391
**Home Guideline-Directed Medical Therapy**				
*β*-Blocker	77% (*n* = 46)	67% (*n* = 14)	82% (*n* = 32)	0.217
ACEi	25% (*n* = 15)	29% (*n* = 6)	23% (*n* = 9)	0.655
ARB	10% (*n* = 6)	5% (*n* = 1)	13% (*n* = 5)	0.269
MRA	30% (*n* = 18)	29% (*n* = 6)	31% (*n* = 12)	0.862
ANRI	17% (*n* = 10)	–	26% (*n* = 10)	–
SGLT2i	10% (*n* = 6)	14% (*n* = 3)	8% (*n* = 3)	0.462
Metoprolol Dose	77 ± 52 (*n* = 39)	77 ± 46 (*n* = 12)	77 ± 56 (*n* = 27)	0.989
Carvedilol Dose	41 ± 16 (*n* = 7)	38 ± 18 (*n* = 2)	43 ± 17 (*n* = 5)	0.768
Lisinopril Dose	15 ± 14	15 ± 14	14 ± 15	0.986
Losartan Dose	29 ± 23	12.5 ± 0	40 ± 37	0.533
Spironolactone Dose	29 ± 23 (*n* = 15)	30 ± 11 (*n* = 5)	29 ± 28 (*n* = 10)	0.903
Eplerenone Dose	33 ± 14 (*n* = 3)	50 ± 0 (*n* = 1)	25 ± 0 (*n* = 2)	–
Entresto Dose	25 ± 0 (*n* = 2)	–	93 ± 63 (*n* = 10)	–
Jardiance Dose	10 ± 0	10 ± 0	10 ± 0	0.998

84% of visits received an IV furosemide bolus, 17% required additional furosemide drip infusions, and 31% required additional metolazone. 5% of patients had mild-moderate hypokalemia, 16% had mild worsening of renal function, and 3% had severe worsening of renal function (Table 2). No hospitalizations occurred due to adverse side effects. No significant changes in potassium, creatinine, and glomerular filtration rate were observed. The mean infusion-visit urine output was 761 ± 521 ml, and post-visit weight loss was −3.9 ± 5.0 kg. No significant differences were observed between HFpEF and HFrEF groups.

**Table 2:** We defined treatment-related adverse events as mild to moderate (serum potassium ≤3.5 mEq/l but >3.0 mEq/l at the first lab draw after the clinic visit, with a decrease of ≥0.5 mEq from baseline) or severe (serum potassium ≤3.0 mEq/l at the first lab draw after the clinic visit, with a decrease of ≥0.5 mEq). Mild worsening of renal function was an increase in serum creatinine ≥0.3 mg/dl but not doubling, while severe worsening was doubling of serum creatinine. (4) Mild-moderate hypokalemia was present in 5% of patients and 3% had worsening renal function. No hospitalizations occurred due to adverse events. Mean urine output: 761 ± 521 ml; mean post-clinic weight loss: −3.9 ± 5.0 kg (* = *p* < 0.001). No significant differences observed between HFpEF and HFrEF groups. A multidisciplinary approach ensured comprehensive care with access to ancillary healthcare for patients.

	Total (*n* = 131)	HFpEF (*n* = 42)	HFrEF (*n* = 89)	*p*-value
**Clinic Intervention**				
IV Furosemide Bolus	84% (*n* = 110)	83% (*n* = 35)	84% (*n* = 75)	0.894
IV Furosemide Infusion	17% (*n* = 23)	17% (*n* = 7)	19% (*n* = 17)	0.735
PO Metolazone	31% (*n* = 41)	33% (*n* = 14)	43% (*n* = 38)	0.304
PO Potassium	29% (*n* = 38)	36% (*n* = 15)	37% (*n* = 33)	0.881
IV Furosemide Bolus Dose	108 ± 25	112 ± 15	106 ± 29	0.182
IV Furosemide Infusion Dose	53 ± 23	53 ± 24	54 ± 23	0.951
PO Metolazone Dose	3.2 ± 1.1	3.8 ± 1.3	3.0 ± 1.0	0.09
PO Potassium Dose	49 ± 19	50 ± 17	48 ± 20	0.576
**Safety**				
Mild-Moderate Hypokalemia	5% (*n* = 5 of 98)	3% (*n* = 1 of 37)	7% (*n* = 4 of 61)	0.359
Mild Worsening Renal Function	16% (*n* = 16 of 98)	19% (*n* = 7 of 37)	15% (*n* = 9 of 61)	0.373
Severe Worsening Renal Function	3% (*n* = 3 of 98)	3% (*n* = 1 of 37)	3% (*n* = 2 of 61)	0.593
**Efficacy**				
Urine Output	761 ± 521	807 ± 604	740 ± 482	0.52
Clinic Weight	261 ± 80	265 ± 64	259 ± 87	0.48
Followup Weight	251 ± 78	250 ± 62	252 ± 86	0.912
Weight Change*	−3.9 ± 5.0	−3.5 ± 4.9	−4.1 ± 5.0	0.559
**Multidisciplinary Care**				
Dietician	8% (*n* = 10)	14% (*n* = 6)	4% (*n* = 4)	0.102
Pallative Care	9% (*n* = 12)	14% (*n* = 6)	7% (*n* = 6)	0.22
Care Management	58% (*n* = 76)	48% (*n* = 20)	63% (*n* = 56)	0.106
Social Work	5% (*n* = 6)	10% (*n* = 4)	2% (*n* = 2)	0.14
Wound Care	11% (*n* = 15)	12% (*n* = 5)	11% (*n* = 10)	0.913

**Figure 2:**
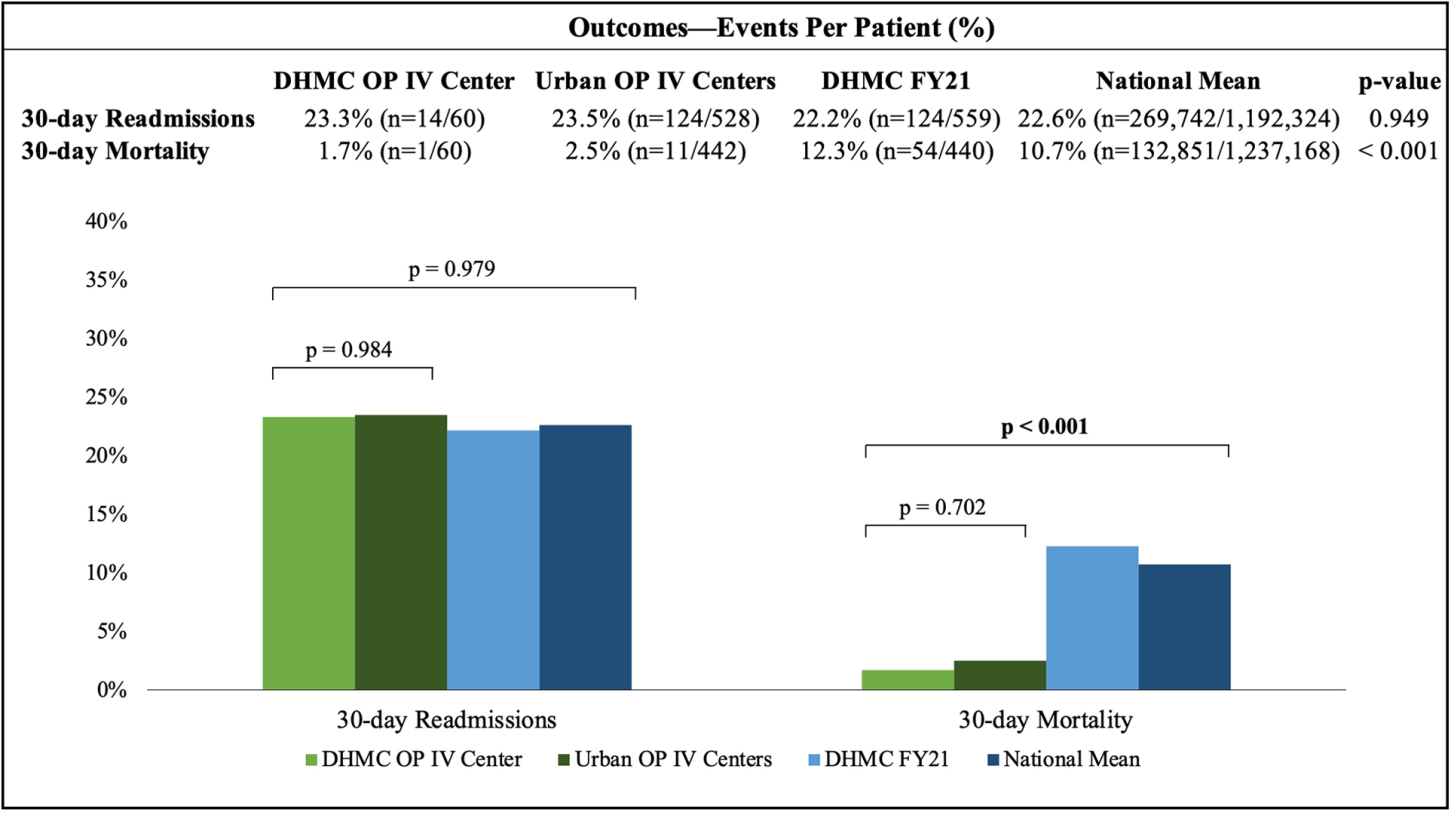
The 30-day readmission rate for the DHMC OP IV center was similar to urban OP IV centers, DHMC FY21, and the national mean. The 30-day mortality rate for the DHMC OP IV center was comparable to urban OP IV centers, but significantly lower than DHMC FY21 and the national mean.

At 30 days, 36% of patients had ≥1 clinic revisit, 42% had ≥1 infusion revisit, 23% were hospitalized, and one death was recorded. At 60 days, 42% of patients had ≥1 clinic revisit, 41% had ≥1 infusion revisit, 33% were hospitalized, and two deaths were recorded. 35% of patients and 16% of visits avoided hospitalizations (Figure 2). 30-day readmission rate was comparable to urban OP IV centers, DHMC FY21, and the national mean (23.3% vs. 23.5% vs. 22.2% vs. 22.6%, respectively; *p* = 0.949). 30-day mortality was comparable to urban OP IV centers, but lower than the DHMC FY21 and national means (1.7% vs. 2.5% vs. 12.3% vs. 10.7%, respectively; *p* < 0.001; Figure 1). A post-hoc sample size analysis was performed on the observed significant differences in 30-day mortality between rural OP IV diuresis and national HF hospitalizations, estimating an attrition of 63 patients for a two-sided alpha of 0.05% and 80% power. Another analysis, comparing rural OP IV diuresis to DHMC FY21, estimated that 112 patients per group would be required for adequate power to observe significant differences in outpatient vs. inpatient management at DHMC.

Per Medicare DHMC FY21 data, HF admission estimated cost was $20,291 (national mean $18,280). The clinic avoided 21 hospitalizations, resulting in estimated cost savings of $426,111. Estimated cost per infusion visit was $990-$1613 ($343 encounter fee, $345 per furosemide bolus, $623 per furosemide drip infusion, $78 basic metabolic panel, and $224 EKG).

## Discussion

To our knowledge, this is the first single-center study to demonstrate the feasibility of providing outpatient IV diuresis in a rural setting. Findings include: (i) DHMC outpatient IV center is safe and effective; (ii) 30-day readmission rate was comparable to urban outpatient IV centers, and inpatient DHMC FY21 and national means. 30-day mortality was significantly lower compared to inpatient DHMC FY21 and national means; (iii) outpatient IV diuresis is an affordable model for rural HF patients.

The safety and efficacy of this study were comparable to those of urban OP IV centers. Transient mild-moderate hypokalemia and reversible episodes of acute kidney injury were observed, with no significant adverse events requiring hospitalization. These findings are consistent with literature, where only a few adverse events were reported in 442 unique patients treated in the reviewed studies, predominantly involving electrolyte disturbances (3–10). A significant mean post-clinic weight loss (-3.9 ± 5.0 kg, *p* < 0.05) was observed. No significant differences were noted in HFpEF and HFrEF groups, although further trials are needed to determine the optimal IV diuretics for use between these two groups (15).

Our clinic's 30-day readmission rate was comparable to urban OP IV centers, DHMC FY21, and the national mean (23.3% vs. 23.5% vs. 22.2% vs. 22.6%, respectively; *p* = 0.949). Our 60-day readmission rates increased to 33%. Although our clinic and other urban centers did not significantly affect 30-day hospitalizations (Figure 2), the 30-day mortality rate for our clinic and urban OP IV centers were significantly lower compared to DHMC FY21 and national mean (1.7% vs. 2.5% vs. 12.3% vs. 10.7%, respectively; *p* < 0.001) (4–7).

Our findings align with Ahmed et al.'s retrospective study, which found no difference in 30-day readmission rates between outpatient and inpatient diuresis (14.9% vs. 13.5%, respectively; *p* = 0.8), but a significantly lower mortality rate in the outpatient group compared to the inpatient group (3.5% vs. 21.3%, respectively; *p* < 0.001). The reduced mortality rates in outpatient IV centers may be attributed to the exclusion of acutely decompensated HF patients and/or the inclusion of close follow-up care compared to hospitalizations. However, a systematic review of five outpatient IV diuresis clinics found limited evidence for improvements in mortality, likely due to limited data and power. Most of these clinics did not compare their results to inpatient groups, making it difficult to determine the effectiveness of outpatient IV diuresis in reducing mortality rates. These conflicting findings highlight the necessity for further research (8, 16).

**Figure 3:**
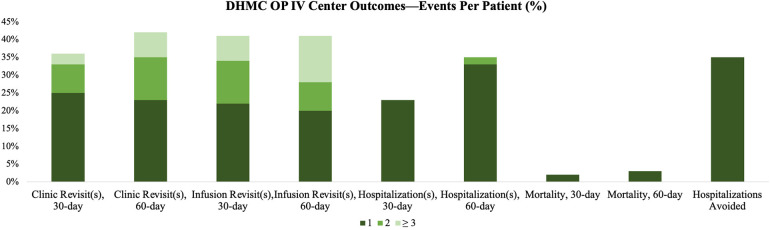
30-day and 60-day clinic and infusion revisits, readmissions, mortality, and the percentage of patients who avoided hospitalization are depicted.

Rural patients often face barriers to accessing healthcare due to higher rates of being uninsured, difficult socioeconomic position, and limited access to care (17, 18). Our study found that the DHMC OP IV center offers a more affordable option with a cost that is one-twentieth of DHMC's FY21 inpatient admission ($990-$1613 vs. $20,291, respectively). We avoided 21 hospitalizations, resulting in a total estimated cost savings of $426,111. Approximately one-third of patients required multiple visits for clinical improvement (Figure 3), which is similar to urban OP IV centers. However, travel times are still a challenge for rural patients (11). 97% of our patients lived in rural areas, with 25% in isolated locations, and had to travel three times more than the average rural American and six times more than the average urban American (18). To address this, we incorporated multidisciplinary resources to provide access to ancillary healthcare needs due to patients' longer travel times, fewer hospitals, and decreased access to specialist care (Table 2).

This pilot study has notable limitations, such as its non-randomized, single-center design, which may introduce selection and measurement biases and uncontrolled confounding factors. In addition, the limited sample size of 60 patients potentially falls short of the estimated 63 needed for a two-sided alpha of 0.05% and 80% power. Prospective data collection may be affected by factors such as funding, staffing, or program awareness. Moreover, the predominantly white rural population studied might not represent the diversity of other rural areas, including higher numbers of Black individuals in the rural South, Hispanics in the rural Southwest, and Indigenous people in the Great Plains (11, 12). Despite these limitations, the study lays the groundwork for a single-center controlled trial at DHMC with a projected minimal sample size of 112 patients per group or larger multi-center trials. Importantly, it offers insights into a transformative model for rural HF patients and may promote the development of similar services in other rural communities.

In conclusion, outpatient IV diuresis appears to be a safe and effective strategy for rural HF patients, with the potential to decrease mortality rates and healthcare costs while mitigating rural-urban disparities. This clinic provides timely and efficient treatment for HF patients without the need for hospitalization. Further research is needed to investigate OP IV diuresis in diverse rural populations, ideally through a randomized, controlled study comparing it to inpatient care.

## Data Availability

The raw data supporting the conclusions of this article will be made available by the authors, without undue reservation.
